# Chylous Ascites and Pleural Transudate: Rare Presentations in Systemic Lupus Erythematosus in Old Age

**DOI:** 10.1155/2012/390831

**Published:** 2012-03-26

**Authors:** Guan-Liang Chen, Deng-Ho Yang, Wen-Hsiu Hsu

**Affiliations:** ^1^Department of Internal Medicine, Taichung Armed Forces General Hospital, Taichung City 411, Taiwan; ^2^Division of Rheumatology/Immunology/Allergy, Department of Internal Medicine, Taichung Armed Forces General Hospital, Taichung City 411, Taiwan; ^3^Division of Gastroenterology, Department of Internal Medicine, Taichung Armed Forces General Hospital, Taichung City 411, Taiwan

## Abstract

Systemic lupus erythematosus (SLE) is a systemic autoimmune disorder with involvement of multiple organs. Various forms of serositis, including pleural effusion, pericardial effusion, and ascites, may be found during the course of SLE. Peritoneal involvement by ascites is common in the initial presentation of SLE. However, chylous ascites is uncommon in SLE patients. Here, we describe a 93-year-old female with initial presentation of chylous ascites during SLE flares. Marked distention and an ovoid shape of the abdomen were observed. Shifting dullness and central tympanic sounds were found on percussion. Rales were heard in bilateral breathing sounds, multiple oral ulcers appeared in the oral cavity, and chest images showed bilateral pleural effusion. Abdominal sonography revealed moderate ascites and pleural effusion. Neither organisms nor malignant cells were revealed in the culture or cytology of ascites and pleural effusion. The diagnosis of SLE was arrived at by positive antinuclear antibody (ANA), discoid rash, oral ulcers, serositis (pleural effusion and ascites), and proteinuria. The patient received intravenous methylprednisolone 250 mg/day for three days. The pleural effusion resolved dramatically after steroid therapy and abdominal distention related to ascites formation subsided obviously.

## 1. Introduction

Systemic lupus erythematosus (SLE) is a systemic autoimmune disorder. It can affect any part of the body, such as the skin, kidneys, joints, liver, lungs, nervous system, and even blood vessels [1]. The damage is thought to be the result of a type III hypersensitivity reaction when an antibody-immune complex attacks our own antigen. As the inflammation cascade starts, cell and tissue damage results. Its clinical course is unpredictable because any type of presentation may develop at any time. Its prevalence is more common in women than in men, especially in women of child-bearing age, and it is more often seen in those of non-European descent. In addition, the clinical course in men and old age is more difficult to recognize, due to vague and rare presentations. 

Various forms of serositis, including pleural effusion, pericardial effusion, and ascites, may be found in the course of SLE. Ascites and pleural effusion have been studied extensively. Sometimes, case reports present chylous pleural effusion. Peritoneal involvement by ascites is not common in the initial presentation of SLE [2]. In fact, ascites in SLE is said to occur only when complicated by nephritic syndrome, congestive cardiac failure, or hepatic cirrhosis [3, 4]. Even then, chylous ascites is not a widely recognized aspect of systemic lupus erythematosus. Only two cases of chylous ascites have been described to date [5, 6]. Here, we present a third case report in this series of clinical presentations.

## 2. Case Report

A 93-year-old bed-ridden woman presented in our emergency department with progressive fullness of the abdomen over two days. She reported no history of alcohol consumption or viral hepatitis. She had just been discharged from the infection ward two days earlier. She was diagnosed with a urinary tract infection with *Proteus mirabilis *and *Klebsiella pneumoniae*. Other symptoms, such as poor appetite, pitting edema of both legs, lethargy, and malaise, were also recorded. On physical examination, a discoid rash on the face and oral ulcers were noticed. There was marked distention and an ovoid shape of the abdomen. Shifting dullness and central tympanic sounds were found on percussion. Sonography of the abdomen revealed moderate ascites and pleural effusion.

Blood count showed a white blood cell count of 7300 cells/uL with 70% neutrophils and 18% lymphocytes, hemoglobin level of 12.0 g/dL, and a decreased platelet count of 112000 cells/uL, and C-reactive protein level was 2.51 mg/dL (normal < 0.5 mg/dL). No obvious deterioration was found in renal function (blood urea nitrogen of 25.2 mg/dL (normal 7–20 mg/dL), creatinine level of 0.95 mg/dL (normal 0.5–1.0 mg/dL)), or liver function (aspartate aminotransferase level of 29 U/L (normal < 31 U/L), alanine aminotransferase level of 22 U/L (normal < 31 U/L)). Immune serological analysis revealed an obvious elevation of antinuclear antibodies (ANAs) 1 : 1280 with a centromere pattern. Levels of other antibodies were all negative. Complement levels were low (C3 45.1 mg/dL (normal 90–180 mg/dL), C4 12.5 mg/dL (normal 10–40 mg/dL)). Immunoglobulin level was within normal limits.

Esophagogastroduodenoscopy revealed gastroesophageal reflux disease, grade A, and erosive gastritis. Computed tomography of the abdomen revealed moderate pleural effusion and ascites. Viral markers for hepatitis C were positive, but no direct evidence of hepatitis or cirrhosis was shown. For further differentiation of fluids in the pleural and abdominal cavity, diagnostic paracentesis was performed. Clear yellow pleural fluid was extracted by thoracocentesis. Pleural effusion analysis showed nucleocytes at 130 cells/uL with 21% neutrophils, 59% lymphocytes, and 12% macrophages; red blood cell count was 450 cells/uL; LDH 87 U/L (serum level 148 U/L); total protein 2.21 g/dL (serum level 6.09 g/dL); glucose 224 mg/dL (serum level 169 mg/dL); PH level 8.0; total cholesterol 34.8 mg/dL. According to Light's criteria, transudate is more likely.

**Figure 1:**
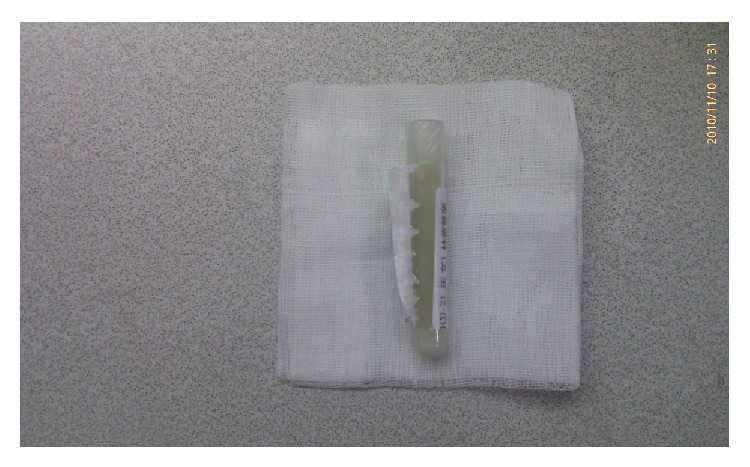
1 milky ascites.

Cloudy and milky ascites were extracted by diagnostic paracentesis (Figure 1). Ascites analysis showed nucleocyte 110 cells/uL with 30% neutrophils, 59% lymphocytes, and 33% macrophages; red blood cell count was 365 cells/uL; albumin 0.24 g/dL (serum level 2.35 g/dL); glucose 315 mg/dL (serum level 169 mg/dL); triglycerides 303 mg/dL. Neither organisms nor malignant cells were revealed in the culture or cytology of ascites and pleural effusion. Chylous ascites was confirmed.

**Figure 2:**
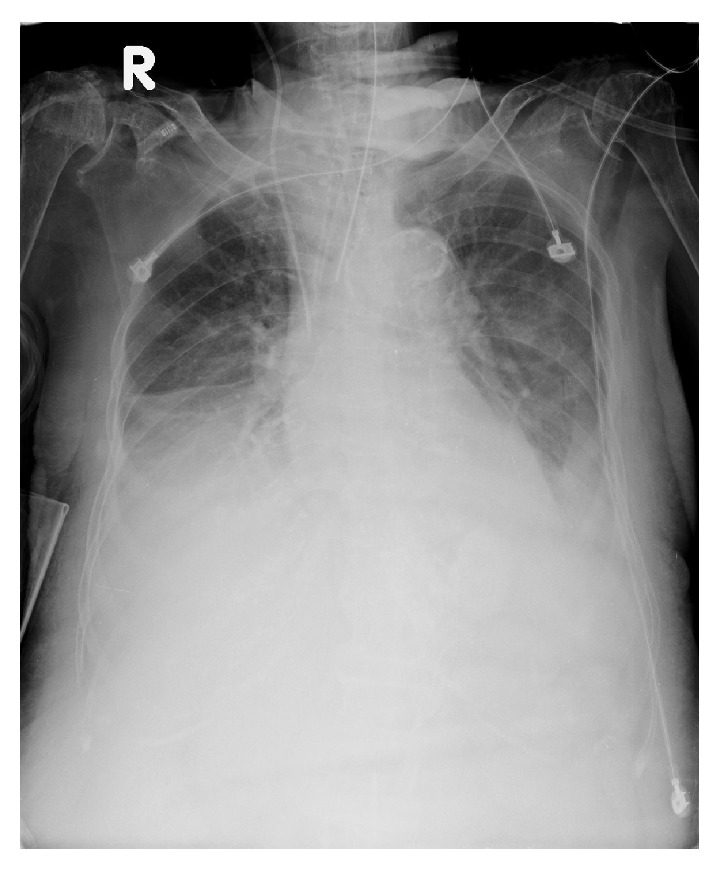
Day 1 on steroid therapy.

**Figure 3:**
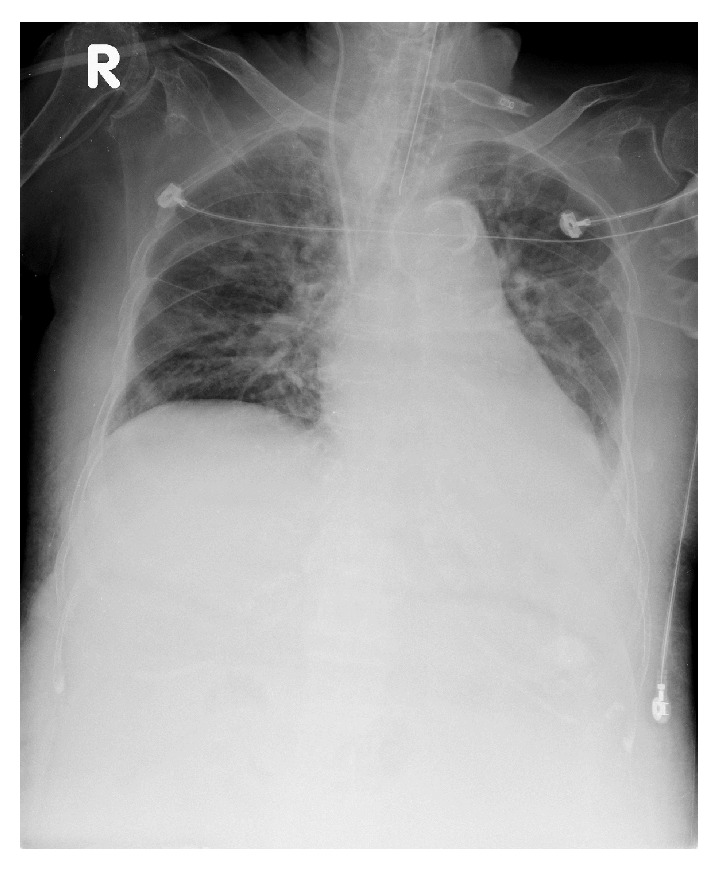
Day 3 on steroid therapy.

The diagnosis of SLE was arrived at by the presence of positive ANA, discoid rash, oral ulcers, serositis (pleural effusion and ascites), and proteinuria (dipstick 2+). Because of the serositis, the patient received 3 days of steroid treatment with intravenous methylprednisolone 250 mg/day. The pleural effusion resolved dramatically after steroid therapy (Figures 2 and 3). Abdominal distention related to ascites formation also subsided obviously. However, because of persistent upper gastrointestinal bleeding and cardiopulmonary distress, the patient died two weeks after admission.

## 3. Discussion

In this case, pleural effusion was analyzed first. Triglyceride levels did not suggest a diagnosis of chylous effusion [7]. The general appearance was clear and yellowish. Pleural effusion frequently develops in SLE patients, and chylous effusion has also been reported. This is not a rare manifestation. By Light's criteria, transudate is favored. Although most pleuritis in SLE is exudate, there are still some case reports of transudate [8]. In the later analysis of ascites, triglyceride levels did suggest chylous ascites. Its general appearance also looked milky. Other causes of chylous ascites were excluded, such as cirrhosis of the liver and malignancy. According to previous case reports, chylous ascites and pleural effusion were considered to be serositis of SLE. Therefore, steroid therapy was prescribed for both forms of serositis that occurred during SLE flares. The patient received intravenous methylprednisolone 250 mg/day for three days. Ascites and pleural effusion resolved rapidly and did not appear again before her death. Later there were persistent upper gastrointestinal bleeding and cardiopulmonary distress. In this case, with combined hepatitis C infection, chylous peritoneal ascites developed as one presentation of SLE, which could be mistaken as a complication of liver cirrhosis. However, ascites and pleural effusion should respond to diuretics rather than to steroids in cirrhotic patients. As a result, clinical improvement after steroid treatment provided additional information that confirmed lupus in this case. The mechanism of SLE causing chylous ascites or chylothorax is still not well understood. The cause might be due to SLE-related inflammation of lymphatic system causing damage, underlying malignancy-induced paraneoplastic syndromes causing obstruction of lymphatic drainage. In summary, chylous ascites is a very rare manifestation of SLE, and it can be the initial presentation of disease course as described in this patient.
